# How to Mitigate the Negative Effect of Emotional Exhaustion among Healthcare Workers: The Role of Safety Climate and Compensation

**DOI:** 10.3390/ijerph18126641

**Published:** 2021-06-21

**Authors:** Mavis Agyemang Opoku, Hyejung Yoon, Seung-Wan Kang, Myoungsoon You

**Affiliations:** 1 College of Business, Gachon University, Seongnam 13120, Korea; opokuagyemangm1@gmail.com; 2 The Seoul Institute, 57 Nambusunhwan-ro, 340-gil, Seocho-gu, Seoul 06756, Korea; yoonhj838@gmail.com; 3 Department of Public Health Sciences, Graduate School of Public Health, Seoul National University, Seoul 08826, Korea

**Keywords:** emotional exhaustion, compensation, safety climate, job satisfaction

## Abstract

This study examines the relationship between emotional exhaustion and job satisfaction. We further propose a safety climate and compensation as contextual variables that weaken the effect of emotional exhaustion. Survey data collected from 694 employees of a public hospital provided support for the hypothesized research model. The hierarchical multiple regression results reveal that high emotional exhaustion is negatively related to job satisfaction. In addition, the results suggest that compensation and a safety climate are moderating variables that mitigate the negative effects of emotional exhaustion. The theoretical implications and future directions are discussed.

## 1. Introduction

Over the past several decades, occupational burnout has garnered the attention of researchers and professionals alike [1]. Burnout is characterized by three dimensions: emotional exhaustion, depersonalization and cynicism [2]. Of these three dimensions, emotional exhaustion represents the stress dimension of burnout [3]. Several studies have provided an insight into the harmful implication of emotional exhaustion at the individual, team and organizational level [4,5]. Given its harmful effects, it is imperative that researchers examine the conditions under which emotional exhaustion can be alleviated or amplified.

The frequency of interaction with customers for service industry workers makes emotional exhaustion prevalent in the service industry [6,7]. For example, service work that requires a significant interaction between employees and customers results in emotional labor as a consequence of the work demand [8]. Emotional labor occurs when frequent interactions with customers are such that workers have to manage their own emotions and those of others in an environment where the emotions displayed by employees are monitored and enforced by management [9]. Within the healthcare industry, these interactions between patients and healthcare providers take on an even more significant meaning because of the varied support that these professionals are expected to offer to their patients [10] while having a caring and empathetic attitude. The emotional toll of taking care of patients makes health service workers more susceptible to emotional exhaustion. Emotionally exhausted employees are less vigilant and more likely to make errors or follow potentially unsafe practices [11]. Within the healthcare industry, emotional exhaustion has been linked to the intention to leave [12] and organizational commitment [13].

The prevalence of emotional exhaustion among healthcare professionals underscores the need to further understand emotional exhaustion within the sector [14]. While much of the literature has examined antecedents, researchers have called for attention to the effects of emotional exhaustion on work outcomes and to identify mechanisms that may reduce its negative effects [13]. Thus, this study examines job satisfaction as a consequence of emotional exhaustion. To do so, this study employs the conservation of resource (COR) theory [15,16,17], which argues that individuals strive to obtain and retain centrally valued resources. When individuals lose resources and become exhausted, they enter a defensive mode to protect against further resource loss, which explains why emotionally exhausted employees are more likely to express a lower job satisfaction [15].

**Figure 1:**
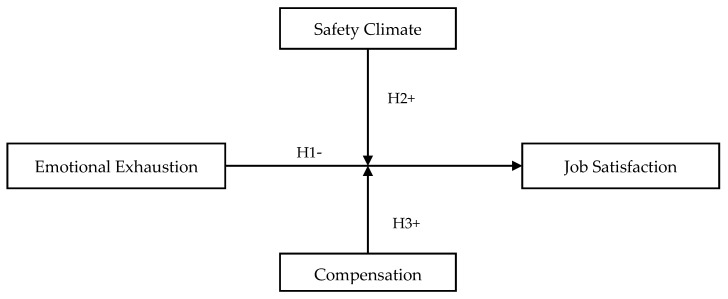
Hypothesized research model.

Additionally, this study investigates two contextual factors that can mitigate the effect of emotional exhaustion. In the event of resource loss, the COR theory proposes two mechanisms of resource investment, namely, resource substitution and resource replacement, that could offset the harmful impact of resource loss. Specifically, we argue that when an organization puts in place measures that promote a safety climate, this would create favorable perceptions of the work environment and could help to lessen the effect of emotional exhaustion. Moreover, we propose that, as a motivational tool, compensation can also play a moderating role. In summary, this study examines the effect of emotional exhaustion and proposes resource investment strategies that can be employed to serve as a buffer against emotional exhaustion particularly in work contexts where exhaustion is prevalent. The hypothesized research model is illustrated in Figure 1.

## 2. Literature Review

### 2.1. Emotional Exhaustion and Job Satisfaction

Emotional exhaustion describes the feeling of being overextended and having one’s emotional and physical resources depleted [2]. Emotional exhaustion reduces the ability of employees to cope with and meet their emotional demands at work [18]. Banks et al. [19] argued that emotional exhaustion hinders the development and maintenance of high-quality exchanges. Thus, emotionally exhausted employees may view the organization through the same lens and interpret the exchanges with the organization as unfavorable. Furthermore, employees who do not feel a strong bond with the social environment at work are more likely to engage in counterproductive behavior such as being rude or hurtful to colleagues at work [20]. In support of this, a meta-analytic review of emotional exhaustion found significant correlations between emotional exhaustion and multiple work attitudes including job satisfaction (*p* = −0.47), turnover intentions (*p* = 0.32) and organizational commitment (*p* = −0.36) [21].

Job satisfaction is indicative of the overall assessment of a person’s work experiences [22]. More specifically, it is an attitude resulting from the evaluative appraisal of three distinct factors: evaluative judgment, affective experience at work and beliefs about the job [23]. Thus, the conceptualization of job satisfaction suggests that emotional exhaustion is an antecedent to job satisfaction. Indeed, Weiss [23] described job satisfaction as an emotional response. As exhausted employees are emotionally and physically depleted, they are more deliberate about how they invest in the limited resources they possess [24]. That is, they would invest in resources that are likely to have a high return rate [15].

Apart from being an affective state, job satisfaction is an affective resource [22]. For example, Ritter et al. [25] explained that experiencing job satisfaction as a resource may be helpful in effectively managing conflict between role sets or preventing resource loss as a function of role conflict. In addition, the accumulation of job satisfaction may enhance the subsequent judgments of employees of role clarity. Given that emotionally exhausted employees have poor quality relationships with peers and, by extension, the organization and are more likely to engage in counterproductive work behavior, we argue that the more emotionally exhausted an employee is, the less likely they are to invest in job satisfaction as a resource. Given that the extant literature provides evidence of this link [26,27], we posit the following:

**Hypothesis** **1.** *Emotional exhaustion is negatively related to job satisfaction.*

### 2.2. The Contextual Role of a Safety Climate

Culture refers to “a pattern of shared basic assumptions that was learned by a group as it solved its problems of external adaptation and internal integration, that has worked well enough to be considered valid and, therefore, to be taught to new members as the correct way to perceive, think, and feel in relation to those problems” [28] (p. 17). Within an organization, the behavior and decisions of employees are guided by the values that underlie organizational culture [29]. In their meta-analytic review, Hartnell et al. [30] presented a comprehensive framework arguing that organizational culture affects several organizational processes and outcomes including employee attitudes and operational and financial effectiveness.

Within the broader context of organizational culture, safety culture is defined as “the set of beliefs, norms, attitudes, roles, and social and technical practices that are concerned with minimizing the exposure of employees, managers, customers and members of the public to conditions considered dangerous or injurious” [31] (p. 4). While organizational culture is assessed phenomenologically through methods such as observations, interviews and mutual comparison, an organizational climate is determined by administering surveys [32]. Thus, this study focuses on a safety climate.

A safety climate is an organizational climate that describes the assessments of employees of the value of safety within the workplace. The components of these evaluations include organizational practices such as the availability of safety equipment and adequate training, management values about employee wellbeing, communication and the level of employee involvement in health and safety protocols [33]. Unlike other forms of organizational climate that are predicated on the perceptions of employees of procedures, practices and rewards, employees can actually experience a safety climate [34]. In hospitals with a strong safety climate, intentional efforts are made to incorporate safety into the daily routines and functions of individuals, work units and organizations as a whole [35].

When the safety climate in the work environment is strong, there tends to be comparatively fewer workplace injuries and higher safety behaviors than those with a weak safety climate [36]. Fewer injuries are not only due to the presence of adequate safety systems but the availability of these systems also signals the commitment of management to employee safety [37]. Previous studies suggest that when hospitals have a strong safety climate, staff tend to place a higher value on patient safety as opposed to productivity or efficiency, thereby reducing the occurrence of medical errors. Additionally, these employees encourage active patient participation in patient safety through actions such as encouraging patients to ask questions and informing patients about abnormal symptoms or allergies [38].

Maslow [39] identified an organization’s ability to satisfy employee needs such as safety as a determinant of job satisfaction. Along these lines, Clarke [40] found that a safety climate is predictive of work attitudes including organizational commitment (r = 0.49) and job satisfaction (r = 0.34). According to the COR theory, when resources are depleted, individuals may employ a resource strategy known as compensation wherein the acquisition of additional resources compensates for the difference between capacity and demand [17]. We argue that under conditions of a high safety climate, this additional resource may compensate for the high emotional exhaustion, which would in turn mitigate the negative effect of emotional exhaustion.

Clark [40] explained that cues that basic safety needs at the job are met would engender positive feelings about one’s work. Thus, even for emotionally exhausted employees, a high safety climate work environment may signal the care and concern of management for employee welfare such that the effect of emotional exhaustion on job satisfaction is weakened. This argument aligns with research detailing that a favorable perception of organizational support engenders positive organizational outcomes [41,42,43]. Empirically, a safety climate has been found to act as a buffer to reduce the negative impact of job demands on safety behavior [44]. Thus, we argue the following:

**Hypothesis** **2.** *A safety climate mitigates the negative effect of emotional exhaustion on job satisfaction such that the negative effect is weakened when the safety climate is high compared with when it is low.*

### 2.3. The Contextual Role of Compensation

An employee’s compensation encompasses direct cash compensation such as salary, indirect non-cash payments such as work benefits, the frequency of salary raises and the processes by which the compensation system is administered [45]. Organizations use compensation to serve multiple purposes: (1) to motivate compliance with organizational rules and regulations [46]; (2) to improve job satisfaction and retention for departments and divisions that are difficult to staff [47] and (3) as a motivational tool to incentivize workers to engage in positive discretionary efforts and behaviors [48]. For employees, compensation symbolizes equity and personal achievement. However, it is not the actual pay or absolute values that are highly correlated with work attitudes and behaviors but rather the perception of an employee of pay [49].

Within the healthcare industry, early scholars viewed healthcare professionals as primarily intrinsically motivated and the role of pay levels played an auxiliary role. Traditionally, the notion has been that these employees are even willing to sacrifice their pay cut in lieu of rewards such as recognition and pride in the ability to express their knowledge and skills [50]. More recently, researchers have continued to discover the benefits of good compensation packages. Pay policies can neutralize the impact of attractive job opportunities at other hospitals [51]. When the compensation expectations of employees are met, they are more committed and driven to meet organizational visions and missions [52], an essential component of successful retention programs [53]. Empirically, there have been inconclusive results on how pay policies influence employee behaviors and attitudes. For instance, while a few studies have found a link between pay levels and turnover [54], others did not find a significant relationship between these variables [55]. Similarly, Shah et al. [56] found a positive relationship between compensation and job satisfaction but other studies did not [57].

Intrinsic motivation and monetary compensation need not be mutually exclusive. As Bowles and Polanía-Reyes [58] explained, intrinsic motivation and monetary rewards interact as substitutes in certain contexts and as complements in other contexts. In Korea, conflict between labor unions and the management of hospitals has been detrimental to employee satisfaction [59]. The bone of contention has been the issue of workload and the level of pay of employees [60]. Thus, we argue that, within the Korean context, compensation may have a stronger predictive effect on employee satisfaction.

The gain paradox principle suggests that under conditions of resource loss, resource gains become even more important [15]. In a comprehensive list of validated resources in western contexts, Hobfoll [16] included numerous finance-related resources such as financial stability, adequate financial credit, savings, money for self-improvement and financial assets. We argue that for emotionally exhausted employees, a favorable perception of one’s compensation may be an avenue to minimize the damaging effect of emotional exhaustion. Based on the theory and extant empirical evidence, we posit the following:

**Hypothesis** **3.** *The perception of compensation mitigates the negative effect of emotional exhaustion on job satisfaction such that the negative effect is weakened when the perception of compensation is favorable compared with when it is not.*

## 3. Method

### 3.1. Sample

Data for this study were obtained from health workers from a public hospital in South Korea. Before the data collection, the researchers visited the hospital to explain the purpose and methodology to the hospital staff. The survey was conducted in Korean after following Brislin’s [61] recommendation for translation. Specifically, two native Korean speakers were tasked to use a translation and back-translation procedure to translate the items from English to Korean. We then conducted a pilot survey for a few employees to obtain feedback on the Korean version instrument [62]. Following the results of the pilot survey, we finalized our questionnaires in Korean. The data collection was conducted with the approval of the Institutional Review Board of the Graduate School of Public Health at Seoul National University (approval number 54-2012-12-31).

The survey packet included a consent form for voluntary participation in the study and was distributed at respective work sites per the number of employees in each ward and department. The respondents were required to submitted completed surveys in a sealed envelope to a designated collection box, which was later collected by the researchers. A total of 1001 questionnaires were distributed, 711 of which were returned. After conducting data cleaning to exclude cases with missing data, a total of 694 responses were used for the analysis.

### 3.2. Measures

All scales were answered on a seven-point Likert scale ranging from 1 (strongly disagree) to 7 (strongly agree) unless otherwise indicated.

#### 3.2.1. Emotional Exhaustion

We used five items from Kalliath et al.’s [63] emotional exhaustion scale for employees reporting their emotional exhaustion. A sample item is: “I feel emotionally drained from my work” (please see Appendix A for the full scale). The Cronbach’s alpha for this scale was 0.91.

#### 3.2.2. Safety Climate

We used a seven-item scale developed by Shteynberg et al. [64] for employees to report their perceptions of the hospital’s safety climate. A sample is: “My suggestions about safety would be acted upon if I expressed them to management” (please see Appendix A for the full scale). The Cronbach’s alpha for this scale was 0.87.

#### 3.2.3. Compensation

We used a two-item scale developed by Hackman and Oldham [65] to have employees report their perceptions of the compensation they receive. A sample item is: “The degree to which I am fairly paid for what I contribute to this organization’’ (please see Appendix A for the full scale). The Cronbach’s alpha for this scale was 0.91.

#### 3.2.4. Job Satisfaction

We used a three-item scale developed by Hackman and Oldham [65] to have employees report the general satisfaction of their job. A sample item is: ‘‘Generally speaking, I am very satisfied with this job’’ (please see Appendix A for the full scale). The Cronbach’s alpha for this scale was 0.91.

#### 3.2.5. Control Variables

We controlled for demographic characteristics including education, organizational tenure, employment status and position to control for possible demographic differences in emotional exhaustion, safety climate, compensation and job satisfaction.

### 3.3. Data Analysis Method

All of the data analyses were performed using STATA 14.1 (Data Analysis and Statistical Software, Stata Corp., College Station, TX, USA). Prior to testing the research model, we conducted a confirmatory factor analysis to verify the discriminant and convergent validity of the measurement model following the Hair et al. [66] cut-off criteria for model fit indices and factor loadings. This included the comparative fit index (CFI) and the Tucker–Lewis index (TLI) ≥ 0.90 and the root-mean-square error of approximation (RMSEA) ≤ 0.08, a normed chi-square ≤ 3.00 with a factor loading ≥ 0.50 and *p*-values ≥ 0.05. An ordinary least squares regression was employed to test the study hypotheses. To limit the possibility of multicollinearity, the study variables were mean-centered and the interaction terms were subsequently created using the centered variables [67].

We conducted a series of regression analyses to test the proposed hypotheses in the study: in step 1, we began by regressing job satisfaction on the control variables; in step 2, we tested the link between emotional exhaustion and job satisfaction role (Hypothesis 1); in step 3, we added compensation, safety climate and the interaction term between emotional exhaustion and the safety climate to test Hypothesis 2; in step 4, we added the interaction term between emotional exhaustion and the safety climate and finally, in step 5, the full model comprised both interaction terms. Following Aiken and West [67], two variables are said to have a significant association when the regression results present a regression coefficient with a significance level that is less than 0.05.

## 4. Results

### 4.1. Characteristics of the Survey Participants

The sample was predominantly female (78%) and 85% of the employees were regular staff, with 15% of them on a contract. Table 1 provides the detailed demographic characteristics of the sample.

### 4.2. Descriptive Statistics and Correlation

The zero-order correlations between the variables and reliabilities are reported in Table 2. As expected, emotional exhaustion had a significant correlation with job satisfaction (*r* = 0.15, *p* < 0.001). Compensation and safety climate also had a significant correlation with job satisfaction (respectively, *r* = 0.57, *p* < 0.001; *r* = 0.61, *p* < 0.001).

### 4.3. Model Validity Check

The hypothesized four factor model produced acceptable model fit indicators, namely, comparative fit index (CFI) = 0.99, Tucker–Lewis index (TLI) = 0.98, root-mean-square error of approximation (RMSEA) = 0.05 and normed chi-square value = 2.6 (χ2 = 275.62, df = 106; normed chi-square = 275.62/102). The factor loadings ranged from 0.68–0.86 (for emotional exhaustion), 0.95–0.97 (for compensation), 0.81–0.87 (for the safety climate) and 0.89–0.98 (for job satisfaction) and were all significant [66,68]. The hypothesized model was compared with three alternative measurement models. All of the alternative models presented weaker model fit indicators and significantly differed from the baseline model as presented in Table 3.

### 4.4. Hypotheses Tests

In support of Hypothesis 1, Table 4 shows that emotional exhaustion had a statistically significant negative relationship with job satisfaction (β = -0.46, *p* < 0.001, Model 2). Hypothesis 2 posited that the safety climate would mitigate the negative effect of emotional exhaustion on job satisfaction. The results from Model 3 showed that the interaction between emotional exhaustion and the safety climate had a positive significant effect on job satisfaction (β = 0.05, *p* < 0.05) while Model 5 showed insignificant results for the interaction (β = 0.01, ns).

**Figure 2:**
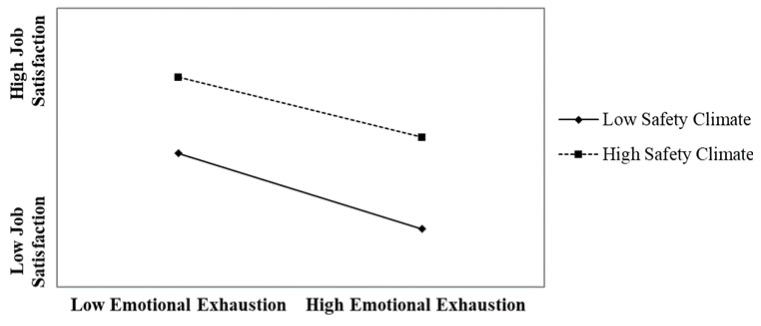
The moderating effect of the safety climate on the relationship between emotional exhaustion and job satisfaction.

Following Aiken and West [67], we plotted the interaction effect using one standard deviation above and below the mean of emotional exhaustion to represent high and low emotional exhaustion, respectively. Figure 2 shows that employees who experienced a lower safety climate experienced even weaker perceptions of job satisfaction when experiencing high emotional exhaustion (slope: β = −0.35, *p* < 0.001). In contrast, employees who experienced a higher safety climate experienced relatively higher perceptions of job satisfaction even when experiencing high emotional exhaustion (slope: β = −0.25, *p* < 0.001). The simple slope difference test (Table 5) revealed that the two slopes were statistically different (*p* < 0.05). Altogether, these results provided partial support for Hypothesis 2.

**Figure 3:**
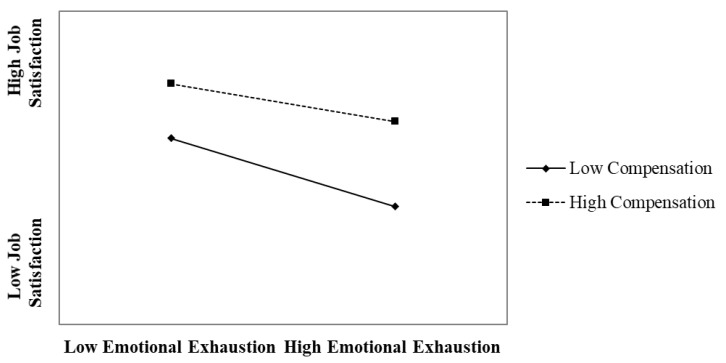
The moderating effect of compensation on the relationship between emotional exhaustion and job satisfaction.

Hypothesis 3 posited that compensation weakens the negative effect of emotional exhaustion on job satisfaction. As shown in the regression results, the interaction between emotional exhaustion and compensation had a positive significant effect on job satisfaction (β = 0.08, *p* < 0.001, Model 4; β = 0.08, *p* < 0.001, Model 5). The simple slope plot in Figure 3 reveals that the employees with less favorable perceptions of the compensation they received experienced even weaker perceptions of job satisfaction when experiencing high emotional exhaustion (slope: β = −0.37, *p* < 0.001). In contrast, the employees with favorable perceptions of the compensation they received experienced relatively higher perceptions of job satisfaction even when experiencing high emotional exhaustion (slope: β = −0.22, *p* < 0.001). The simple slope difference test revealed that the two slopes were statistically different (*p* < 0.001). The regression and simple slope tests supported Hypothesis 3.

## 5. Discussion

### 5.1. Theoretical Implications

This study sought to make several theoretical contributions to the emotional exhaustion literature. This research reinforces previous empirical studies linking emotional exhaustion to multiple employee attitudes and behaviors in the workplace [4,5]. Specifically, this study was conducted within the context of the public healthcare sector where the characteristics of the work environment often contribute to the prevalence of emotional exhaustion [69]. Using the COR theory, we argued that because emotionally exhausted individuals are emotionally and physically depleted, they are less likely to invest limited resources in job satisfaction, which is an effective resource. Not surprisingly, we found emotional exhaustion to be negatively related to employee job satisfaction.

The most important contribution of our study lies in identifying the moderating variables in this relation. This is because much of the research has focused on the direct effect of emotional exhaustion but not much is known about the job resources or organizational variables that may serve as a buffer against the damaging effects of emotional exhaustion [69]. In addressing this gap in the literature, we developed and tested a research model that examined two conditions under which the effects of emotional exhaustion could be mitigated at the individual level. Basing our model on the premise that certain job resources can unleash motivational processes, the empirical results provided partial support for the hypothesized research model suggesting that there were resources that an organization could employ when dealing with emotionally exhausted employees to minimize the effect on job satisfaction.

We found marginal support for the hypothesis that the relationship between emotional exhaustion and job satisfaction was mitigated by a safety climate. As a safety climate is directly experienced by employees, when policies and practices that promote a safe working environment for both employees and patients are instituted, this can make it easier to cope with emotional exhaustion. Even though a safety climate may require an increased workload for employees, it points to the commitment and prioritization of employee safety of hospital management. Given that exhausted employees have fewer opportunities to establish positive work experiences [69], organizational support for a high safety climate can be an avenue to provide such experiences.

Moreover, when employees have a favorable perception of the compensation received for work done, even for emotionally exhausted employees the effect of emotional exhaustion on job satisfaction is weakened. While early researchers considered healthcare professionals to be principally intrinsically motivated, emerging research points to the importance of compensation to commitment and a reduced turnover [52,53]. Compensation is a motivational tool used to incentivize employees [48]. We expected compensation to take on even more significance for emotionally exhausted employees because it would represent an acknowledgment by superiors of the toll that the job demands engendered. The study findings were consistent with the COR theory, which has previously suggested that emotional exhaustion can be reduced if an individual’s resources are increased [70]. By extending the COR theory, this study moved beyond the moderating role of psychological resources [70] and pointed to other resources that could serve as a safeguard against emotional exhaustion.

Although the study did not hypothesize the main effect of compensation and safety climate, the results suggested that both mechanisms had a positive effect on job satisfaction. Regarding safety climate, this finding suggested that the effects of a safety climate go beyond traditional accidents and safety outcomes to influence other human resource outcomes. The results of a positive significant relationship between the perception of compensation and job satisfaction suggested that even for industries that are traditionally conceptualized as being intrinsically motivated, a favorable perception of the salary received is key in keeping employees satisfied.

### 5.2. Managerial Implications

The results of this study have multiple implications for managers. First, the results reinforce previous studies by which emotional exhaustion has a negative association with employee attitudes and behaviors. Hence, it is important for organizations to pay more attention to and identify and develop organizational strategies and interventions that address emotional exhaustion among healthcare professionals. This is important because emotional exhaustion affects multiple outcomes including patient safety, work engagement and turnover, for example. Alternatively, given that healthcare is a high-stress industry, hospitals may want to recruit employees with personality traits such as a high emotional stability who can withstand the emotional toll of day-to-day activities.

This research suggests that in situations where emotional exhaustion cannot be addressed a safety climate may also be employed to mitigate the harmful effects of emotional exhaustion. Similarly, for the issue of compensation, which is a constant source of conflict between employees or unions and management, organizations would need to take a nuanced look at compensation and understand its implications for business strategy and planning. Particularly for a very demanding work context, management may use compensation to improve perceptions of job satisfaction.

### 5.3. Limitations and Future Studies

The study results should be interpreted with the following limitations in mind. First, the data for all study variables were derived from the same source. This use of self-report cross-sectional data precludes causal interpretations of the findings [71]. Although the factor structure analysis points to the distinction between the four constructs and an adequate fit of the data with the measurement model, future research must employ more rigorous research designs to validate the study results and allow for a more definitive interpretation. Longitudinal studies would shed more light on the causal links. For instance, it would be beneficial to understand how changes in compensation affect the main effect relationship.

Second, this research treated a safety climate as an individual-level construct to understand how individual perceptions of the climate affect employee attitudes. However, the organizational climate reflects shared beliefs among organizational members about organizational practices and procedures [72] and is best treated as a group-level variable. Thus, future research should employ a multilevel methodology.

Finally, future research must examine other possible moderators of the relationship between emotional exhaustion and job satisfaction. Given the crucial role leadership plays in the workplace [73,74,75,76], it would be beneficial to expand the research model by including the role of leadership. Potential moderators could also include personality traits such as extraversion or agreeability, job characteristics such as role clarity or ambiguity and team dynamics including team member exchanges. Although it was important to test the research among hospital staff to allow for generalizability within the healthcare sector, it would be relevant to unearth the differences that exist between different professionals or groups. For instance, are there differences between medical and non-medical staff or between contract and regular staff with regard to the research model?

## 6. Conclusions

Drawing on the COR theory, the current research reinforced the extant literature and showed that emotional exhaustion was a predictor of low job satisfaction. The added value of this study lay in identifying the mechanisms that could reduce the damaging effects of emotional exhaustion. This approach provided important insights as there are certain industries where emotional exhaustion cannot be entirely eradicated. The findings suggested that when employees believed that the safety climate in the work environment was high, the negative effect of emotional exhaustion on job satisfaction was reduced. Additionally, the favorable perception of employees of the compensation received served as a buffer to reduce the effects on emotional exhaustion. Despite its limitations, this research has made important theoretical contributions to the emotional exhaustion literature as well as practical interventions for field managers.

## Figures and Tables

**Table 1:** Demographic characteristics of the sample.

Variable	Percent
Gender	
Male	21.76
Female	78.24
Age	
20–29	38.04%
30–39	34.58%
40–49	18.73%
50–59	8.51%
60–69	0.14%
Education	
High School	6.20%
2–year College	38.18%
4–year College	41.79%
Masters	11.38%
Doctoral	2.45%
Employment	
Regular Staff = 1	85.01%
Contract Staff = 2	14.99%
Organizational tenure (in years)	
<1	8.36%
1–2	29.68%
3–4	19.45%
5–8	13.26%
9–12	6.92%
13–16	4.03%
17–20	7.06%
21–24	6.34%
>25	4.90%
Department	
Medical	9.22%
Nurse	53.46%
Administration	10.09%
Healthcare (Ex. physical therapists and radiologists)	18.73%
Research	1.44%
Others	7.06%
Position	
Employee = 1	79.54%
Deputy leader = 2	7.64%
Team leader = 3	3.46%
Manager or higher = 4	9.36

**Table 2:** Descriptive statistics and inter-correlations.

	Mean	SD	1	2	3	4	5	6	7	8
1. Education	2.66	0.85	−							
2. Tenure	3.82	2.28	0.08 *	−						
3. Employment (regular staff)	1.28	0.74	−0.04	0.21***	−					
4. Position	1.66	1.64	−0.22 ***	0.19 ***	−0.26 ***	−				
5. Emotional exhaustion	4.45	1.26	−0.11 **	−0.17 ***	0.20 ***	−0.20 ***	(0.91)			
6. Compensation	3.05	1.56	0.02	0.25 ***	−0.13 ***	0.08	−0.40 ***	(0.96)		
7. Safety climate	4.83	1.11	−0.02	0.35 ***	0.03	0.00	−0.28 ***	0.46 ***	(0.95)	
8. Job satisfaction	4.61	1.34	0.13 ***	0.34 ***	−0.08 **	0.16 ***	−0.52 ***	0.57 ***	0.61 ***	(0.95)

Note: n = 694. Cronbach’s alpha values for each scale are reported in parentheses on the diagonal. Employment was coded as regular staff = 1 and contract staff = 0. * *p* < 0.05, ** *p* < 0.01, *** *p* < 0.001 (two-tailed).

**Table 3:** Model fit statistics for the measurement models.

Measurement Model	χ^2^	Df	CFI	TLI	RMSEA	Δχ^2^	Δdf
Baseline (hypothesized) four factor model	275.62 ***	106	0.99	0.98	0.05		
Alternative 1 (three factor model) ^1^	1335.56 ***	109	0.89	0.86	0.13	1059.94 ***	3
Alternative 2 (two factor model) ^2^	2642.64 ***	111	0.78	0.72	0.18	2367.02 ***	5
Alternative 3 (one factor model) ^3^	3879.72 ***	112	0.67	0.59	0.22	3604.10 ***	6

Note: n = 694. *, *p* < 0.05, **, *p* < 0.01, *** *p* < 0.001 (two-tailed test). ^1^ Three factor model with compensation and satisfaction on the same factor. ^2^ Two factor model with emotional exhaustion, safety climate and compensation on the same factor. ^3^ Three factor model with emotional exhaustion, safety climate, compensation and job satisfaction on the same factor.

**Table 4:** Hierarchical multiple regression for job satisfaction.

Variables	Model 1	Model 2	Model 3	Model 4	Model 5
Education	0.09	0.06	0.09 **	0.08 **	0.08 **
Tenure	0.36 ***	0.27 ***	0.09 **	0.08 **	0.08 **
Employment (regular staff)	−0.15 ***	−0.05	−0.00	−0.01	−0.01
Position	0.04	−0.01	0.05	0.05	0.05
Emotional exhaustion (EE)		−0.46 ***	−0.28 ***	−0.27***	−0.28 ***
Safety climate (SC)			0.38 ***	0.38***	0.38 ***
Compensation (CO)			0.26 ***	0.27***	0.27 ***
**Interaction**					
EE x SC			0.05 *		0.01
EE x CO				0.08 **	0.08 **
F	31.17 ***	71.47 ***	117.07 ***	119.08 ***	105.71 ***
R^2^	0.15	0.34	0.57	0.58	0.58
Adj R^2^	0.14	0.33	0.56	0.57	0.57
F_inc_		197.20 ***	127.40 ***	130.93 ***^a^	0.06

Note: n = 694. Employment was coded as regular staff = 1 and contract staff = 2; employee position was coded as employee = 1, deputy leader = 2, team leader = 3 and manager or higher = 4. Entries are the standardized regression coefficients. ^a^ = F-test compared with Model 2. * *p* < 0.05; ** *p* < 0.01, *** *p* < 0.001 (two-tailed test).

**Table 5:** Simple slope test.

Moderator	Safety Climate	Compensation
Low level of moderator	−0.35 ***	−0.37 ***
High level of moderator	−0.25 ***	−0.22 ***
Simple slope difference test	0.10 *	0.15 **

* *p* < 0.05; ** *p* < 0.01, *** *p* < 0.001 (two-tailed test).

## Appendix A

Emotional Exhaustion (the Cronbach’s alpha for this scale was 0.91) [63]:

1. I feel emotionally drained from my work.
2. I feel fatigued when I get up in the morning and have to face another day on the job.
3. I feel frustrated by my job.
4. I feel I’m working too hard on my job.
5. I feel like I’m at the end of my rope.

Safety Climate (the Cronbach’s alpha for this scale was 0.87) [64]:

1. The culture of this hospital makes it easy to learn from the mistakes of others.
2. Medical errors are handled appropriately in this hospital.
3. I am encouraged by my colleagues to report any patient safety concerns I may have.
4. I know the proper channels to direct questions regarding patient safety.
5. I receive appropriate feedback about my performance.
6. I would feel safe being treated here as a patient.
7. My suggestions about safety would be acted upon if I expressed them to management.

Perception of Compensation (the Cronbach’s alpha for this scale was 0.91) [65]:

1. The amount of pay and fringe benefits I receive.
2. The degree to which I am fairly paid for what I contribute to this organization.

Job Satisfaction (the Cronbach’s alpha for this scale was 0.91) [65]:

1. Generally speaking, I am very satisfied with this job.
2. I am generally satisfied with this kind of work I do in this job.
3. I frequently think of quitting this job (R).
